# Mefloquine and Its Enantiomers Are Active against *Mycobacterium tuberculosis* In Vitro and in Macrophages

**DOI:** 10.1155/2014/530815

**Published:** 2014-12-11

**Authors:** Luiz E. Bermudez, Laura Meek

**Affiliations:** ^1^Department of Biomedical Sciences, College of Veterinary Medicine, Oregon State University, 105 Magruder Hall, Corvallis, OR 97331, USA; ^2^Department of Biomedical Sciences, College of Sciences, Oregon State University, 107 Dryden Hall, Corvallis, OR 97331, USA

## Abstract

*Objective*. Tuberculosis is a serious problem of public health. The increase on the number of clinical cases of tuberculosis infected with multidrug resistant (MDR) *M. tuberculosis* calls for the development of novel therapy. *Design*. We investigated the effect of mefloquine and two enantiomers, (+)erythro-mefloquine and (+)threo-mefloquine against *M. tuberculosis* strains in the environment resembling the aspects of the granuloma environment and in macrophages. *Results*. The results suggest that mefloquine (racemic mixture) and (+)erythro-mefloquine have bactericidal activity against *M. tuberculosis* strains both in acidic, low oxygen tension and in macrophages. The activity, however, was impaired under increased osmolarity. *Conclusion*. Identification of the target for mefloquine in the pathogen will allow for the development of novel drugs with antituberculosis activity.

## 1. Introduction

Despite tuberculosis being a serious human condition, associated with high mortality, the treatment of the disease has evolved very little over the years. The chief therapy for the disease is still based on antibiotics introduced more than 40 years ago. Recently, new compounds have shown promising activity in animal models and are currently in clinical trials [1, 2]. In spite of that, the need of novel targets against which new antibiotics can be developed is pressing. For example, TMC207 (bedaquiline, a diarylquinoline) is a compound with a novel target in* M. tuberculosis* [2, 3], the subunit C of the proton pump for ATP synthase. ATP synthase is an enzyme that utilizes the flow of protons across the bacterial membrane to synthesize ATP. As such, the compound is very attractive, but there is a high probability that resistance would develop very quickly, since bacteria have several mechanisms for ATP synthesis. In addition, spectinamides modified to provide antituberculosis activity may indicate an effective approach for the development of novel antituberculosis compounds [4]. Meropenem, a *β*-lactam antibiotic that inhibits the L-D transpeptidase, also has been shown to achieve against* M. tuberculosis* [5] and the class of *β*-lactams (carbapenems) is being increasingly explored. *β*-lactams are safe antibiotics and therefore desirable as an option.

Testing a series of quinolines for antibacterial activity against* Mycobacterium avium*, we identified a potent, both in vitro and in vivo, bactericidal compound, mefloquine [6, 7]. Although mefloquine is commonly prescribed as prophylaxis against malaria, due to the short duration of the prophylaxis, its antimycobacterial activity was never appreciated. Clinical use of mefloquine in a small number of patients with disseminated and pulmonary* Mycobacterium avium *infection has been reported showing success [8]. In addition, mefloquine can be part of a three drug regimen that does not involve macrolides, for* M. avium* disease as demonstrated in an experimental model of the infection [9].

Based on the above described findings, we decided to investigate the activity of mefloquine against* M. tuberculosis. *During the course of the infection in humans,* M. tuberculosis* lives under the environments encountered in the phagocyte vacuole and in granulomas.

In fact, in both environments the pathogen is exposed to conditions such as low oxygen tension, hyperosmolarity, and acidic pH [10]. While in macrophages,* M. tuberculosis* inhibits the acidification of the phagosome [11]. Later in the course of the infection the bacterium is able to survive in the acidic environment encountered in the caseum. Therefore, compounds with antituberculosis activity need to be versatile and have activity against the bacterium under diverse environmental conditions. This property is not always common to the compounds used to treat tuberculosis and many of them have limited activity to phase-of-disease and lacking maximal activity in some of the conditions encountered during infection.

We determined years ago that mefloquine, a quinoline, had bactericidal activity in vitro and in vivo against organisms of the* Mycobacterium avium* complex [6, 7]. Mefloquine is a compound approved for the treatment and prophylaxis against malaria [12] and has a very long half-life which allows administration once a week. Studies in mice have indicated that the compound may be administered every three days for the treatment of disseminated* M. avium* infection [7]. In addition, mefloquine achieves very high concentration within cells, a property desirable for antituberculosis compounds.

Mefloquine is an enantiomer, made of (+)erythro-mefloquine, (−)erythro-mefloquine, (+)threo-mefloquine, and (−)threo-mefloquine. In this study we evaluated the activity of mefloquine and its enantiomers against different strains of* M. tuberculosis* under aerobic and anaerobic conditions, in hyperosmolar conditions, and under acidic pH. The active enantiomers were bactericidal in all the conditions used, although they showed slightly diminished activity in hyperosmolar environment.

## 2. Materials and Methods

### 2.1. Bacteria

*M. tuberculosis* H37Rv and Erdman were obtained from American Type Culture Collection and were cultured onto Middlebrook 7H10 agar plates with OAC (oleic acid, albumin, and catalase, Difco Detroit, MI) at 37°C. Colonies were checked for purity and placed in 7H9 broth with OAC for 5 days. Then, the suspension was centrifuged (1,000x rpm) and the pellet resuspended in Hank's balanced salt solution (HBSS). The concentration was then adjusted to the desired number of organisms using McFarland standard. Two different clinical isolates obtained from China (isolate 400) and from Brazil (isolate 368), resistant to INH and rifampin, were also used.

### 2.2. Antibiotics

The racemic mixture of mefloquine was provided by the Walter Reed Army Institute for Research (Bethesda MD). The enantiomers (erythro and threo) were produced from the racemic mixture as described [13]. All compounds were obtained from the Walter Reed Army Institute for Research under NIH contract N01-AI-5402.

INH and rifampin were purchased from Sigma Chemicals (St. Louis, MO).

### 2.3. In Vitro Testing

The minimal inhibitory concentration (MIC) was performed as previously described [6]. The inoculum was prepared to 10^5^ organisms (confirmed by plating onto 7H10 agar with OAC). The controls included bacteria without adding drug. The bacterial growth was monitored daily and the results were recorded after 20 days.

To determine whether mefloquine was active under condition of restricted oxygen tension, we tested the H37Rv and Erdman strains using the Wayne system [14]. Mefloquine (+)erythro-mefloquine and (+)threo-mefloquine were evaluated against bacteria in nonreplicating state. Concentrations from 2 *μ*g/mL to 128 *μ*g/mL were used.

In addition, hyperosmolar and acidic conditions supplementing 7H9 broth with dextrose at equimolar concentrations (0.3 M) and pH 5.5 (established using 0.1 N HCl) were used as environments in which the activity of mefloquine was examined. Bacteria were maintained under the described condition for 24 hs and then exposed to mefloquine, erythro-mefloquine, and threo-mefloquine.

### 2.4. Macrophage Assay

The activity of mefloquine and its enantiomers were also determined using the macrophage model. THP-1 macrophages were seeded as 10^5^ cells in a 24-well and incubated with 5 *μ*g/mL of phorbol-myristate acetate (PMA, Sigma, St. Louis, MO). After 12 hours, adherent cells were washed and then incubated with 1 × 10^5^ H37Rv for 1 h. Following phagocytosis, macrophage monolayers were washed with HBSS to remove the extracellular bacteria. The monolayer was then treated with different concentrations of mefloquine and enantiomers for 4 days, when then the monolayers were lysed as previously described [6]. Control wells were run in parallel to ensure lack of drug-associated toxicity. Macrophage lysates were then diluted and plated onto 7H10 agar plates for quantification of the number of intracellular viable bacteria.

### 2.5. Statistical Analysis

The significance of the result was analyzed using the Student's test and one-way analysis of variance (ANOVA). A *P* value equal to or below 0.05 was considered statistically significant.

## 3. Results

### 3.1. MIC In Vitro

To determine the susceptibility of* M. tuberculosis* isolates to mefloquine and enantiomers in vitro, bacterial strains were exposed to different concentrations of mefloquine, (+)threo-mefloquine, (−)threo-mefloquine, (+)erythro-mefloquine, and (−)erythro-mefloquine.  Table 1 shows the MIC for two multidrug resistant strains of* M. tuberculosis*, as well as for H37Rv and Erdman strains. The results indicate that mefloquine and (+)erythro-mefloquine are more active against H37Rv and Erdman* M. tuberculosis* strains than (−)erythro-mefloquine, (+)threo-mefloquine, and (−)threo-mefloquine. When tested against MDR strains, the results were similar to the ones obtained against H37Rv and Erdman strains, demonstrating that the compound does not have cross-resistance to Rifampin and INH.

### 3.2. Activity under Environment of Decreased Oxygen Tension

The environment in which many* M. tuberculosis* bacteria are encountered in vivo is poor in oxygen [10]. Because a number of antituberculosis drugs lose activity in an oxygen-depleted environment, we carried out the determination MIC assay under microanaerobic environment (nonreplicating bacteria, NRB). It was determined that mefloquine and both (+)erythro-mefloquine and (−)erythro-mefloquine had similar MIC to the one obtained in an environment with oxygen (Table 2).

### 3.3. Activity under Hyperosmolar Environment and Acidic pH

Because the environment in the granuloma is hyperosmolar and acidic, characteristics that can impact on the susceptibility of the microorganism to the antimicrobials, bacteria were incubated in both conditions for 24 hs and then exposed to mefloquine or enantiomers. As shown in Table 3, exposure to mefloquine under increased osmolarity (0.3 M of dextrose) but not under acidic pH affected the ability of the compound to inhibit* M. tuberculosis* growth. When mefloquine was tested at 0.1 M concentration of dextrose, the MIC obtained was 8 *μ*g/mL for H37Rv and Erdman strains, demonstrating that the effect of hyperosmolarity on the susceptibility was concentration dependent.

### 3.4. Activity in Macrophages

To examine the activity of mefloquine and enantiomers against intracellular* M. tuberculosis*, we carried out macrophage infection with H37Rv strain of* M. tuberculosis* and treated infected monolayers with 4 *μ*g/mL of the compound. The concentration of 4 *μ*g/mL is the serum concentration of mefloquine. The compound is known to concentrate within erythrocytes and hepatocytes up to 80-fold [12]. Mefloquine and the erythro enantiomers were significantly active against intracellular H37Rv (Table 4). No toxicity on the monolayer was observed.

## 4. Discussion

Mefloquine is a quinoline used for the prophylaxis of malaria. The medication is administered once a week to individuals that travel through malaria-endemic regions. The finding that mefloquine has bactericidal activity against* M. tuberculosis* and* M. avium* complex is remarkable and supported by the fact that another quinoline, TMC207 (bedaquiline), is active against* M. tuberculosis* as well [2, 3, 6]. The observation, however, that exposure to bedaquiline results in the selection of resistant mutants while the exposure to mefloquine is not associated with the development of resistance against the compound [15] suggests that either the target(s) on the bacterium is (are) not the same or mefloquine has more than one target on mycobacteria.

In this study, we examined the activity of mefloquine and enantiomers against* M. tuberculosis*. Similarly to what has been observed with* M. avium* [6, 7] both mefloquine and (+)erythro-mefloquine are the most active compounds while the threo-enantiomers showed poor activity. We also evaluated the activity of the compounds under conditions encountered within granulomas. One of the limitations of antituberculosis therapy is that not all the used antibiotics are equally active under both aerobic and anaerobic conditions. Because the environment inside the granuloma is microaerophilic [10] a new compound should be able to perform well under those conditions. In fact, under anaerobic environment of the granuloma,* M. tuberculosis* does not replicate (NRC) which makes the majority of antibiotics that have as target the replication machinery (DNA), not active or with impaired activity. The finding that mefloquine and (+)erythro-mefloquine are active against* M. tuberculosis* under anaerobic condition suggests that the target for the compounds is not in the bacterial replication machinery. We also evaluated* M. tuberculosis* under acid pH and hyperosmolar conditions.

Mefloquine as well as (+)erythro-mefloquine was active at acidic pH. This characteristic of the drug is also important, because only pyrazinamide among the antituberculosis compounds is totally active under acidic conditions that are encountered in the caseum [15]. One of the interesting questions regarding the cavitary granuloma is why* M. tuberculosis* survives in the acidic pH in the granuloma but spends energy to inhibit the fusion of the lysosome with the phagosome in macrophages. The explanation can be multifactorial, but one possibility is that* M. tuberculosis* probably behaves differently when in the acidic conditions of the caseum or in the phagosome.

The assays also showed that when the MIC was obtained in hyperosmolarity conditions, a different response to the antimicrobials was detected. Although less active in hyperosmolar environment, both mefloquine and (+)erythro-mefloquine are likely to achieve high, bactericidal concentrations in the caseum. Studies with* M. avium* in mice suggest that the compounds are effective in killing bacteria in the lung, liver, and spleen lesions [6, 7]. Differently from the* M. tuberculosis* granuloma in mice, which does not undergo necrosis, the* M. avium* granuloma liquefies with caseum formation [16].

Mefloquine is also active in macrophages, despite the extracellular concentration of the drug being only 4 *μ*g/mL, whereas the in vitro MIC has been determined to be 8 *μ*g/mL. These findings suggest that mefloquine and (+)erythro-mefloquine can concentrate inside the phagocytic cells, which is a desirable feature in any antituberculosis compounds.

Studies with* M. avium* indicate that (+)erythro-mefloquine has increasing activity against intracellular bacteria, compared with mefloquine. This is also true for* M. tuberculosis* is unknown at this point.

In summary, our study demonstrated that mefloquine an (+)erythro-mefloquine has antituberculosis bactericidal activity. Mefloquine has side effects associated with the central nervous system (CNS) in humans [17]; however, (+)erythro-mefloquine appears to be less associated with it [7]. The discovery of the target(s) for mefloquine may allow for the design of compounds with antituberculosis activity, but less associated with CNS side effects.

## Figures and Tables

**Table 1:** Mefloquine and enantiomers MIC against two strains of MDR *M. tuberculosis* and H37Rv and Erdman strains. Results in mcg/mL.

Compounds	Strains			
	H37Rv	Erdman	MDR Mtb	
			Strain 368	Strain 400
Mefloquine	8	8	8	16
(+)Threo-mefloquine	16	16	16	32
(−)Threo-mefloquine	32	32	32	64
(+)Erythro-mefloquine	8	8	8	16
(−)Erythro-mefloquine	8	8	8	32

**Table 2:** Activity of mefloquine, (+)erythro-mefloquine, and (−)erythro-mefloquine against H37Rv, Erdman and MDR-TB strains in oxygen-depleted environment (NRB) in vitro (Wayne model).

Bacteria strain	MIC (mcg/mL)		
	Mefloquine	(+)Erythro-mefloquine	(−)Erythro-mefloquine
H37Rv	8	8	8
Edman	8	8	8
MDR Mtb 368 (Brazil)	8	8	8
MDR Mtb 400 (Asia)	8	8	8

Conditions obtained using the Wayne system.

**Table 3:** Activity of mefloquine and (+)erythro-mefloquine in conditions of hyperosmolarity and acidic pH.

MIC (mcg/mL)						
	Mefloquine			(+)Erythro-mefloquine		
	7H9	7H9 pH 5.5	7H9 0.3 M	7H9	7H9 pH 5.5	7H9 0.3 M
H37Rv	8	8	32	8	8	32
Ederman	8	8	16	8	8	16

Bacteria were incubated with antibiotic in 7H9 broth, 7H9 + pH 5.5, and 7H9 + 0.3 M of dextrose.

**Table 4:** Mefloquine and enantiomer activity against H37Rv in macrophages. Serum level of mefloquine (4 *µ*g/mL).

Compound	CFU/mL of lysate	*P* value
No treatment time 0	3.0 ± 0.5 × 10^5^	—
No treatment day 4	1.7 ± 0.3 × 10^6^	—
Mefloquine (4 *µ*g/mL)	5.9 ± 0.4 × 10^4^	<0.05
(+)Erythro (4 *µ*g/mL)	3.4 ± 0.3 × 10^4^	<0.05
(−)Erythro (4 *µ*g/mL)	6.1 ± 0.5 × 10^4^	<0.05
(+)Threo (4 *µ*g/mL)	5.1 ± 0.3 × 10^5^	>0.05
(−)Threo (4 *µ*g/mL)	7.4 ± 0.5 × 10^5^	>0.05

THP-1 macrophages: 1 × 10^5^.

Monolayers treated daily for 4 days. No toxicity observed.

Results of 3 distinct experiment (mean ± SD).
